# “When in Rome…”: structural determinants impacting healthcare access, health outcomes, and well-being of South Asian older adults in Ontario using a multilingual qualitative approach

**DOI:** 10.3389/fpubh.2024.1405851

**Published:** 2024-12-17

**Authors:** Diya Chowdhury, Catherine Tong, Kimberly Lopez, Elena Neiterman, Paul Stolee

**Affiliations:** University of Waterloo, Waterloo, ON, Canada

**Keywords:** healthcare system, structural determinants, access to care, cultural competence, intersectionality

## Abstract

With the increase in international migration, the need for an equitable healthcare system in Canada is increasing. The current biomedical model of healthcare is constructed largely in the Eurocentric tradition of medicine, which often disregards the diverse health perspectives of Canada’s racialized immigrant older adults. As a result, current healthcare approaches (adopted in the US and Canada) fall short in addressing the health needs of a considerable segment of the population, impeding their ability to access healthcare services. This study aimed to identify and understand the structural and systemic factors that influence healthcare experiences and well-being among South Asian older adults in Ontario, addressing a significant gap in empirical and theoretical knowledge in the Canadian context. We conducted in-depth individual and dyadic interviews (n = 28) utilizing a descriptive multilingual cross-cultural qualitative approach. Through this research, participants expressed that their understanding of well-being does not align with that of their healthcare providers, resulting in unmet health needs. Our study uses an intersectional lens to demonstrate participants’ perceptions of virtual access to care and systemic factors, such as mandatory assimilation and whiteness as a taken-for-granted norm impacting the health and well-being of South Asian older adults. The findings of this research can offer valuable insights to healthcare providers and policymakers in developing culturally competent practices, guidelines, and training policies that effectively address the healthcare needs of the South Asian population in Canada.

## Introduction

There has been considerable research and policy attention related to the needs of the aging population in Canada, but older adults still face significant obstacles when it comes to accessing services, which remains a prominent challenge for this subgroup (1). The presence of ethnocultural diversity among the older adult population further complicates the situation in terms of both access and equity (2). Historically, Canada and other Global North nations have been structured in a manner that primarily benefits the majority of the population (3). However, it is also important to acknowledge that people identifying as white receive more attentive treatment over racialized individuals, since the entire system has been constructed with a bias towards prioritizing the needs and interests of people with the greatest proximity to whiteness (4–7). Consequently, it is unsurprising that individuals who are less proximal to whiteness encounter additional challenges in having their healthcare needs met (8). Some communities of older adults are currently grappling with fundamental issues, such as access to care, that are essential for their overall welfare (3). In particular, migrants from diverse cultural backgrounds who are racialized are especially vulnerable. Being an older adult has disadvantages in a youth-oriented society, and these disadvantages may be exacerbated for older adults who are also members of a racialized ethnocultural group, putting their well-being at greater risk (3, 9). Racialized immigrant older adults may face a higher likelihood of receiving inadequate support from health professionals within their new home nation (10–13). This lack of support appears to stem from their encounters with barriers to care, exposure to discrimination, and unequitable treatment within the Canadian healthcare system (13–15).

## Background

In the sections below, we discuss access to care, the need for an intersectional lens, the intersection of white normativity, capitalism, and patriarchy, the need for anti-racist perspectives, and cultural competence and humility as they relate to the health care experiences of racialized immigrant older adults in Canada.

### Defining access to care

The effectiveness of healthcare systems in Canada and around the world depends largely, but not solely, on access to care (16). In the last century, there have been significant variations in the definitions and interpretations of healthcare access (16–20). Different authors’ interpretations of *access to care* demonstrate the complexity of the concept (21, 22), though, access in the context of health care is generally characterised by the ability or simplicity with which consumers or communities are able to use relevant services in proportion to their needs (21, 23, 24). Opinions regarding the components of care access vary and it is generally undecided whether the emphasis should be on describing provider characteristics or the actual process of care; despite that, the term “access” is frequently used to describe factors or characteristics influencing the first point of contact with services or the use of services (25, 26).

Penchansky & Thomas (16) were among those who defined care access in terms of the compatibility of client expectations and client attributes with provider characteristics and health services. In this context, access may be perceived as the point of contact between potential patients and healthcare resources, and it would be impacted by both the qualities of people who provide the services and those who utilize them (16). According to Mooney (19), access depends on both supply and demand. Based on this perspective, the location, cost, appropriateness, and availability of services and burden of disease are products of supply variables. On the other hand, Frenk (17) provides a theoretically appealing way to view access which is to think of it as the degree of adaptation between the population’s characteristics and those of the healthcare resources. This perspective sees access as a functional relationship between the public, healthcare facilities, and resources that reflects the existence of either barriers or facilitators for the recipients of healthcare (17). Following a thorough analysis of the terms used to describe health care access, Levesque et al. (23), proposed defining access as a set of opportunities to “identify healthcare needs, to seek healthcare services, to reach the healthcare resources, to obtain or use health care services, and to actually be offered services appropriate to the needs for care” (p4). This is the definition adopted for the study at hand.

### Intersectionality

The intersectionality movement emphasises the significance of looking at the various, interconnected systems of dominance that influence and shape people’s lives through the interaction of differentiating categories, such as race, sex, and age, within larger systems of oppression (27–31). Intersectionality, coined by Crenshaw (29), was borne of a statement by a Black feminist lesbian organization that criticized the widespread exclusion of Black women from the anti-racist, white feminist movements of the time (29, 32, 33). The idea that social categories are organised through systems of dominance, which are the historically rooted structures of racism, patriarchy, and capitalism, all of which stem from colonization, is at the heart of this literature (34). Thus, intersectionality scholars assert that every process of differentiation and system of dominance is interconnected, where oppressive and dominating structures could not exist or function without racial and gender inequality, economic exploitation, sexism, heterosexism, and other forms of discrimination (28).

Impacts of social inequities are felt by people as simultaneous interactions between many identity markers that are embedded within larger systems of power, dominance, and oppression (35–37). Each individual’s intersecting identities, social positions, and the larger socio-political contexts in which they exist are specifically relevant to the experience of discrimination, access to power, and resources/privileges such as positive health-care experiences (38, 39). Moreover, the essential premise of intersectionality is the recognition that individual’s experiences differ noticeably from others because of the multiplicity and variety of social location, identity, and many forms of inequality they encounter (40). It highlights the fact that there are political and social repercussions to the categories of differences of age, gender, and race and that they are mutable (40). For instance, given the variety of experiences and identities that go under the umbrella term “South Asian,” it is incorrect to assume that just because women are members of a homogeneous racialized group such as South Asians, their experiences will be the same (41). In light of this, intersectional theory highlights what is overlooked when a certain identity is essentialized as the defining quality of a group (by highlighting race, for instance), and the other internal identities, such as gender, age, or citizenship/legal status, are excluded (41, 42).

### The intersectional oppression of the big three: white normativity, patriarchy, and capitalism

Sociologist Mary Romero highlighted a significant divide between immigration research and the sociology of race in her article published in Contemporary Justice Review in 2008, expressing that there exists a substantial ideological and theoretical gap between these two fields (43). Since then, numerous publications have worked towards bridging this divide by integrating race analysis and shifting away from assimilationist frameworks. For instance, Sanchez and Romero (44) emphasize the significance of race in shedding light on issues of illegality and racialized citizenship. Sáenz and Douglas (45) demonstrate how assimilationist frameworks may mask the impact of racial antagonism towards immigrants. Treitler (46) further elucidates how assimilationist viewpoints reinforce how white normativity places the burden of “integration”/adopting the dominant culture on racialized immigrants while overcoming or disregarding racial barriers. Recognizing the interconnectedness of various systems of inequality such as patriarchy, capitalism, and white normativity is crucial. Patriarchy, white normativity, and global capitalism are interconnected systems of oppression that significantly influence migration patterns and the process of immigrant integration (47, 48). Patriarchy refers to a social structure where men occupy positions of power and authority in various realms such as the family, community, government, and society at large (49, 50). white normativity entails the dominance and control exerted by white individuals or groups (51). Global capitalism aims to maximize profits for capitalists by exploiting workers, which is an inherent aspect of the capitalist system (52). By employing an intersectionality framework, we emphasize the simultaneous influence of these oppressive systems on the lives of racialized immigrants (30, 43, 53–56).

### The need for anti-colonial and anti-racist perspectives in examining and reframing healthcare

Although the history of the start of colonization dates farther back, the early 19^th^ century’s newly conceived theories of evolution gave way to the white normativity that exists today (5). European biomedical systems operated under the believe that white populations were the “evolutionarily-selected beings” while racialized individuals were the “non-evolved, lack of the human” (57). One of the ways in which oppression and mistreatment of the “other, lesser/non-human” was rationalized by this belief and white superiority was assumed and reinforced by legal, healthcare, and education systems (5). Despite modern science rejecting the idea of a single classification system for assigning race, the social and political significance of race and racialization remains an important consideration (58–60). In other words, “race may not be real, but racism is” (61). For instance, after the Railway construction in Canada, Chinese immigrants were categorised as one of the lesser “non-white” races compared to white individuals (5, 57). Furthermore, in Canada, the genocide of Indigenous peoples and the exploitation of people of African descent are rooted in structural racism from its colonial past (62–64).

Since contemporary inequities of racial discrimination are related to historic global colonization, an anti-colonial perspective can aid in challenging the racism rooted in colonialism (62, 65, 66). An anti-colonial perspective is concerned with resistance against imperial and colonial ideologies/practices, while upholding equality and justice to re-establish Indigenous forms of knowledge and culture that reflects and centres around the global majority (66, 67). Complementary to this, an anti-oppressive perspective works to address systems of domination that “denies individuals dignity, human rights, social resources and power” (68). Similarly, an anti-racist perspective involves examining the power imbalances between racialized and non-racialized persons and addressing racism within other “interlocking systems of oppression” (69, 70). While frameworks of anti-racism and anti-oppression share overlapping conceptual and theoretical features, the difference lies in the fact that the latter does not predetermine a particular category of oppression, whereas the former analyzes privilege, power, and oppression through the lens of race (62, 71–73). Employing anti-racism and anti-colonial frameworks on hegemonic approaches to healthcare can provide strategies to challenge institutional power dynamics, interrupt production of social inequities within medicine of the Global North and enable a deeper understanding on how racism and colonialism, even still, shapes and structures the Canadian healthcare system (65, 74).

### Cultural competence and cultural humility

Cultural competence is increasingly recognized as a framework to improve health outcomes and satisfaction with care for racialized and ethnoculturally diverse populations produced by migration (75–79). Cross et al. (80) provide a commonly used definition of cultural competence that entails allowing systems/individuals to operate effectively and respond respectfully in cross-cultural contexts (12). However, given the critiques of cultural competency [see (81–83)], scholars have suggested replacing cultural competence with cultural humility (84–87). Cultural humility entails addressing power imbalances between physician and patient, by committing to self-reflection and critiquing internal biases to develop mutually constructive, non-paternalistic relationships (88, 89). Cultural humility expands the traditional considerations of ethnicity and race to include the multidimensionality of cultures of individuals from diverse backgrounds (88). By taking cultural fluidity into account, cultural humility is attentive to the mutability of other social-identity indicators, such as sexual orientation and gender (90). While a key strength of cultural humility is that it does not necessitate providers to be experts on cultural knowledge of care-recipients, several scholars argue that cultural humility lacks conceptual clarity and a concrete definition (81, 89, 91, 92). Another fundamental critique of cultural humility is the assumption that being culturally humble equates to having respect for diversity (85, 90, 93, 94).

While most of the literature dichotomizes cultural competence and cultural humility, it can be argued that both constructs can be used complementarily by offsetting mutual deficits and enhancing mutual strengths. Both cultural competence and humility emerged as a response to the ethnocultural diversity in nations of the global majority and the need for implementing an anti-colonial and anti-racism framework in healthcare contexts (77, 95). Therefore, instead of directing attention to the differences between the two, a better approach proposed in the literature is to find mutuality, recognize why the constructs emerged in the first place, and focus on attaining outcomes (i.e., address inequities) that one cannot achieve without the other (78, 96). In alignment with cultural humility practices, providers need to embrace culture as fluid and complex, avoid essentializing care-recipients, and approach patients from a “not-knowing” position to equalize the power dynamics at play (89, 96). Similarly, in accordance with cultural competence, providers must recognise and overcome cultural prejudices and forge relationships that transcend cultural barriers (82, 97, 98).

### The present study

Despite being one of the largest and most rapidly growing racialized communities in Canada, there exists a knowledge gap pertaining to the health needs and experiences of South Asian communities (99). Various studies have indicated that South Asian individuals born in Canada tend to encounter worse health outcomes when compared to their Canadian-born counterparts (100–108). The data on South Asian older adults in Canada is limited, with a focus on areas such as mental health, specific chronic diseases, and some socioeconomic factors (36, 109, 110). Consequently, there is a noticeable gap in both empirical and theoretical knowledge concerning the health and well-being of South Asian older adults in the Canadian context, particularly regarding the structural determinants that influence their health and well-being at the individual level. The purpose of our study was to identify and understand the structural determinants and systemic factors influencing the healthcare experiences and well-being of South Asian older adults in Canada. While the study initially set out to understand individual-level experiences, participants largely pointed towards structural and systemic issues which, both directly and indirectly, impacted their healthcare experiences and overall well-being. The study also provides recommendations in alignment with a solutions-oriented perspective to facilitate culturally competent care delivery, aiming to foster positive health outcomes.

## Methods

The present study and analysis form a component of a broader qualitative inquiry which sought to understand the involvement or lack thereof of racialized immigrant older adults in their own healthcare (111). To understand the perspectives of and experiences with the healthcare system among South Asian older adults residing in Canada, who were born in foreign countries, we conducted 28 in-depth qualitative interviews. These interviews were conducted in six different South Asian languages, utilizing a descriptive qualitative approach that was both multilingual and cross-cultural (112). The chosen methodology for this study is qualitative description (113). This study was carried out in the Southern region of Ontario, Canada, which is a significant hub for immigration and home to diverse communities. We acknowledge that the term “South Asian” may oversimplify and overlook the lived experiences and individual identities of people within this group. Therefore, instead of relying solely on country of origin, we recruited participants based on the languages they spoke. By adopting this approach, we aimed to identify cultural groups connected through language, rather than being limited by arbitrarily defined borders imposed by colonization. It is important to note that while the selected languages represent the most commonly spoken languages in South Asia, this methodology allowed us to better reflect shared cultural experiences.

### Study, setting, and context

South Asians constitute 25% of the racialized population in Canada and make up 5.6% of the overall population (114). Toronto, the capital of Ontario, and its surrounding region, known as the Greater Toronto Area, is home to significant concentrations of South Asian immigrants, with a substantial number coming from countries such as India, Pakistan, Bangladesh, and Sri Lanka (115). The majority of individuals of South Asian origin in these areas are foreign-born (68%), while the remaining 32% were born in Canada (114).

### Sample, eligibility, and recruitment

Participants in the study were older adults (aged 60 years and above) of South Asian descent, presently living in Southern Ontario. They were required to be capable of giving informed consent and conducting the interviews in one of the following languages: Hindi, Punjabi, Tamil, Urdu, Bangla, or English. Since we focused on an immigrant population, we utilized the World Health Organization’s age cut-off of 60 for defining older adults, which aligns with how the term “older adult” has been utilized in previous studies involving individuals from South Asia (116, 117). Participants were recruited from various South Asian community organizations using a combination of methods such as email and community referrals. Initially, some of the participants who had already joined the study voluntarily helped with snowball sampling, following the approach outlined by (118). They shared our recruitment materials with their friends and family who also met the criteria for participation. In preparation for the interviews, consent forms were distributed in advance through email. Further information can be found in the protocol paper by Tong et al. (111), that outlines the methods of the larger study in detail.

## Data collection and analysis

This study received ethics clearance from the University of Waterloo Office of Research Ethics (ORE#43297). Data collection took place September 2020 to December 2021.

### Qualitative interviews

Data for this study were collected during the on-going COVID-19 pandemic in Canada. Due to the prevailing circumstances and restrictions imposed on in-person interactions, all interviews were conducted remotely via video calls, providing flexibility for participants to choose a convenient time. A total of 28 South Asian older adults participated in the study; information regarding participants’ demographics can be found in Table 1. The determination of the sample size was guided by the concept of information power proposed by Malterud and colleagues (119), considering the richness of the obtained data and the availability of relevant theories within the existing literature. Among the participants, 12 requested dyad interviews, and 16 completed individual one-on-one interviews. Although the same interview guide was used for both interview types, the wording of the questions was adjusted accordingly. The dyad interviews were intentionally extended to ensure the inclusion of all the questions asked to each participant. To protect participant confidentiality, pseudonyms were assigned during the analysis, and Table 2 provides links between the pseudonyms and demographic information, interview language, and interview type.

**Table 1:** Participant demographics.

Interview sample (*n* = 28)	
Age	Mean: 74
	(min: 60, max: 88)
Gender	
Men	13
Women	15
Country of origin	
India	11
Bangladesh	6
Sri Lanka	7
Pakistan	4
Years in Canada	Mean: 34 (min: 10, max: 62)
Education	
Secondary	3
Undergraduate	14
Graduate	11
Marital status	
Married	25
Widowed	1
Divorced	2
Lives with	
Spouse	8
Spouse & children	14
Spouse, children, & grandchildren	3
Alone	3
Has family doctor	
Yes	26
No	2
English Proficiency (self-reported)	
Reading	
Poor	1
Well	2
Very well	25
Writing	
Poor	0
Well	2
Very well	26
Speaking	
Poor	2
Well	0
Very well	26

**Table 2:** Interview participant pseudonyms and demographic information.

Pseudonym	Age	Gender	Country of origin	Religion/Faith system	Length of time in Canada (Years)	Type of interview conducted	Language interview conducted in
Ashish	88	Man	Bangladesh	Hinduism	29	Individual	English
Rajib	74	Man	Bangladesh	Hinduism	12	Dyad	Bengali
Aarini	62	Woman	Bangladesh	Hinduism	15	Dyad	Bengali
Prisha	63	Woman	India	Hinduism	51	Individual	Hindi
Shivanya	76	Woman	India	Hinduism	51	Individual	Tamil
Shankar	86	Man	India	Hinduism	52	Individual	English
Zarina	60	Woman	Pakistan	Islam	17	Individual	Urdu
Irfaan	68	Man	Pakistan	Islam	21	Dyad	Urdu
Aaron	75	Man	India	Christianity	57	Dyad	English
Aileen	68	Woman	India	Christianity	52	Dyad	English
Prithvish	71	Man	India	Hinduism	45	Individual	Tamil
Shastik	77	Man	Sri Lanka	Hinduism	26	Dyad	Tamil
Gayatri	78	Woman	Sri Lanka	Hinduism	31	Dyad	Tamil
Sharvaa	73	Woman	Sri Lanka	Hinduism	25	Dyad	Tamil
Abhijeet	75	Man	Sri Lanka	Hinduism	45	Dyad	Hindi
Tarala	78	Woman	Sri Lanka	Hinduism	28	Individual	English
Sarath	82	Man	Sri Lanka	Hinduism	28	Dyad	English
Srijit	75	Man	Bangladesh	Hinduism	48	Individual	Bengali
Amit	86	Man	India	Hinduism	53	Individual	English
Shalini	67	Woman	Sri Lanka	Hinduism	42	Dyad	Hindi
Jamal	60	Man	Pakistan	Islam	16	Dyad	Urdu
Beena	73	Woman	India	Hinduism	34	Individual	English
Ananya	61	Woman	India	Hinduism	32	Individual	Hindi
Janaia	64	Woman	India	Hinduism	10	Individual	English
Sharad	87	Man	India	Hinduism	62	Individual	English
Shujata	77	Woman	Bangladesh	Hinduism	34	Individual	Bengali
Malini	83	Woman	India	Hinduism	10	Individual	Punjabi
Ravi	72	Man	India	Hinduism	45	Individual	Hindi

A semi-structured interview guide was utilized to explore participants’ experiences with and perspectives of the healthcare system. Sample interview questions can be found in Table 3. The interviews had an average duration of 84 min, ranging from 32 to 120 min. All interviews were recorded and transcribed digitally. As a token of appreciation for their time, interview participants received an honorarium in the form of a $20 CAD gift card.

**Table 3:** Sample interview questions.

Primary questions	Probe
Have you ever disagreed with your healthcare provider and/or their decisions regarding your health?	If so, could you tell me how you dealt with that?
When you want to know more about your health, where do you go for information?	Family, internet, news, social media, talking with friends, relatives overseas, etc.
What role do family members play in your health care?	Do they join you for appointments? Drive you to appointments? Do they help with translation? Do they get medications for you? Prepare special meals? What does this support mean to you?
What would you like health care providers to know/understand about your culture?	How does that impact your health?

The challenges associated with interpreting and translating qualitative data have been acknowledged by qualitative researchers (120). It is important to note that translating qualitative data goes beyond linguistic conversion and involves incorporating the cultural context in which the data were collected (121). Given the anticipated complexity and multilingual nature of our data collection, we employed the written translation approach outlined by Inhetveen (120) to translate the interviews into English and generate English-language transcripts for analysis. DC and Research Assistants who are fluent in the South Asian languages used in the study carried out the translation. This approach, performed shortly after the interviews, ensured a comprehensive translation that captured all details and aimed to achieve a lexical equivalence with the original phrasing (120). Following the guidelines proposed by McKenna (122), we also adopted a forward translation method, whereby the original source language was translated into the language used for analysis and reporting. Consequently, all transcripts were translated into English, allowing for comprehensive analysis of the dataset (122).

### Theoretical framework: multilevel eco-social framework

The “socio-ecological model” was developed in the late 1970s to acknowledge the extensive array of social influences and nested environmental interactions affecting individuals (123). This model is built upon various levels of environmental impacts, with the individual and their unique characteristics at its core (123, 124). The first level of influence, known as microsystems, includes factors such as family, spiritual systems, and healthcare systems (123). The interactions and dynamics between two or more microsystems constitute the second level of influence, referred to as mesosystems (e.g., family and health clinic interactions) (124). Moving up, the next level of influence is exo-systems, encompassing larger social systems comprising two or more settings and both direct and indirect influences (e.g., caregiver leave eligibility) (124). Finally, the topmost level, called macrosystems, involves broad cultural and subcultural characteristics that exert an impact on all the other layers (e.g., attitudes and belief systems of the larger culture) (124).

The multilevel eco-social framework [see (13)], builds upon the socio-ecological model and delves into the intricate relationship between racialized older immigrants and their access to healthcare. This framework views healthcare access as a complex social process, influenced by a combination of individual and contextual factors that interplay between the health needs of older immigrants and healthcare providers (13). Bronfenbrenner’s ecological theory also contributes to this understanding by proposing that seeking healthcare among racialized immigrants involves a person-environment interaction across various overlapping systems, leading to an ecological transition in healthcare (123).

### Qualitative analysis and strategies for rigor

The transcripts underwent a series of preparatory steps, including cleaning, anonymization, and uploading into NVivo 12 along with field notes and meeting notes, for analysis. Braun and Clarke’s (125) thematic analysis approach was selected as the analytical method for examining the data in this research. In addition to the theoretical flexibility that Braun and Clarke’s (125) thematic analysis offers, while preserving data complexity, this approach also emphasizes the active involvement of the researcher in knowledge production. Our coding process followed an iterative approach, involving the proposal of initial codes and the subsequent addition of new codes as required. Concepts from Lin’s (13) multilevel eco-social framework guided the initial coding structure. This iterative process was carried out through a line-by-line analysis of the data, led by the first author DC. A detailed account of the iterative coding strategy can be found in Table 4. To ensure rigor in our analysis, we engaged in member checking as well as reflexive memoing during the coding process (126), holding regular team meetings throughout the data collection and analysis phases (127), and conducting team-based analysis involving members from different disciplines and ethnocultural backgrounds.

**Table 4:** A priori and emergent, evolving coding structure.

Initial codes from the multi-level eco-social framework	Codes added during further reading of transcripts	Codes less salient, either removed or collapsed; not highlighted in the results	Final, high-level clustering of themes and sub-themes, as presented in the results
Exo-system: Labour & capital adequacy Language services Sensitivity Professional values	Virtual care Healthcare equity	Appointment system Navigation Impacts of covid-19	Access to Care The importance of language services Virtual care and its role in enabling or hindering access
Macro-system: Healthcare policy Immigration policy Societal values and ideology Oppression and discrimination Stigma	Cultural Humility and competence Holistic vs. biomedical model of healthcare White normativity	Long term care services	Cultural Humility and Competence Culture competence for mental health needs Holistic versus the biomedical model of care Making space for “other” ways of being, healing, and care
	Need to fit into the system		Negotiating Candidacy The need to be a “good” citizen Eurocentrism and “whiteness” as a standard

## Results

Through the analysis we identified three themes and respective sub-themes pertaining to structural determinants and systemic factors influencing the healthcare experiences and well-being of South Asian older adults in Canada, summarized in Table 5. The first theme of access to care highlighted the need for language services to be embedded within the healthcare system to cater to racialized immigrant older adults that have language barriers. The results also demonstrate how virtual care both enabled and hindered access to care. The second theme underscored the importance of culturally competent and humble care delivery, while also highlighting the challenges that arise with a lack of cultural competence and humility in healthcare provision. Within this theme, the findings highlighted the importance of incorporating alternative methods of understanding and healing into the existing biomedical-focused healthcare model. The results demonstrate that this adaptation is necessary to effectively address the needs of patients and to ensure healthcare providers have a comprehensive understanding of their patients’ broader care context beyond the formal healthcare system. Lastly, the final theme of our analysis, negotiating candidacy, indicates that participants have felt a pressure to conform to societal norms that have historically been set by white individuals and upheld by white individuals, racialized individuals, and the overarching system.

**Table 5:** Summary of themes and sub-themes.

Theme	Sub-theme(s)
Access to care	The importance of language services
	The skills and resources needed to access virtual care
Cultural competence and humility	The need for cultural competence and humility in healthcare provision
	Holistic versus biomedical model of care
Negotiating candidacy	The need to “fit in” and whiteness as the standard

### Access to care

To reiterate, in our study, we adopted Levesque et al., ‘s (23) definition of access, which encompasses a comprehensive set of opportunities: “to identify healthcare needs, to seek healthcare services, to reach the healthcare resources, to obtain or use health care services, and to actually be offered services appropriate to the needs for care” (p. 4).

#### The importance of language services

Participants highlighted the difficulties they faced as a result of language barriers, as well as those faced by their relatives and social connections, such as recent immigrants. The problems associated with language underscored how language barriers impeded access to healthcare. As an example, one participant, Irfaan, discussed the significance of having a physician who shares the same language as them,

*Initially we got a Chinese doctor who was good but couldn’t understand us well. So, we had to find an Urdu speaking doctor. It’s very hard for us Pakistanis without someone who can speak Urdu. I can’t speak in English very well at all. My children can understand English very well, but they can’t come with me every time* (Irfaan, male, 68 years old).

Other participants have also expressed a similar sentiment regarding the significance of involving family members instead of relying on formal language services. Jamal highlighted the challenges they encounter when trying to buy medications because of the obstacles posed by language differences,

*It would be impossible for me to go get medications if my son or daughter didn’t go with me. Everything is in English, and I am not a fluent speaker. Even with my doctor, who speaks Urdu with me, gives me the prescriptions in English, right? Thankfully, the kids know English and go with us. I don’t know what we would do without them* (Jamal, male, 60 years old).

This quote highlights the dependence that many participants have on their children and family members to access and utilize the healthcare system. Additionally, this emphasizes that even if patients and their healthcare providers share the same language, there may still be a lack of language concordance with other members of the healthcare team who may not speak the patient’s language. Consequently, the significance of language concordance extends beyond the patient’s primary point of contact, typically their family physician, as there are other crucial healthcare professionals within the system who play important roles in patient care. A few participants specifically brought up the need for translation services and how that would be helpful for those with language barriers and a lack of familial support. On this point, Ravi noted,

*It’s hard for people who don’t know English that well. They don’t come from an educated background, so how would they know English? My children can go with me if I need them to, but some of our relatives here from India, who would they go to the doctor with? Their kids don’t live close because their workplace is far from here. If they have some, some kind of translator at the hospital or the walk-in clinics, that would help, right? You know, there’s lot of Indians here, they can at least provide the basic languages for the immigrants here* (Ravi, male, 72 years old).

Ravi’s statement also highlights education as a potential determinant impacting a patient’s healthcare experience, indicating that individuals with lower education might encounter greater challenges in accessing the healthcare system. Participants also extensively discussed the scarcity of available doctors and the limited access to specialized care, underscoring the prolonged wait times within the existing healthcare system.

#### The skills and resources needed to access virtual care

The COVID-19 pandemic led to the more rapid adoption of technology, as provinces sought virtual care as a means of ensuring safe access to healthcare services. Participants in the study shared varied opinions regarding the usefulness and user-friendliness of virtual care. Some participants acknowledged its utility, highlighting its conveniences. Shankar, talks about his perspectives on virtual care and the benefits of technology,

*No. I didn’t face any challenges with virtual services. It is all accessible. And, you know, we are equipped for it, so amazing. I mean, technically, the computers are after our time, right, we have not well advanced as far as, you know, use of the computer, but we can still make use of it. We are not like, like my sister. She's in India. She's only four years older than I am. But she won't touch the computer* (Shankar, male, 86 years old).

Shankar’s quote underscores the notion that for certain individuals, in order to participate in virtual care and consequently gain access to healthcare services through virtual care, there is a need for them to adjust and familiarize themselves with using technology. However, other participants expressed concerns about the challenges faced by their family members who are recent immigrants or those who are less proficient in English, suggesting that virtual care may not be as accessible or beneficial for them. One such quote is provided by Prisha,

*Covid-19 was actually quite helpful for us. My doctor is far, so talking to them online means I don’t have to travel that far to speak with him. And I don't feel it’s necessary to speak with my doctor face to face. But I can see it being a challenge for people like my sister who just came to Canada not long ago. Her English isn’t very strong so I can imagine it being much harder for her* (Prisha, female, 63 years old).

Other participants pointed out that virtual care was especially beneficial for individuals living with disability. Tarala describes her situation with her father-in-law, who has limited mobility, and how virtual care had an impact on their experience,

*This [virtual care] is a godsend because it's very difficult to take my father-in-law in the clinic with the catheter, and all that we have to change into a leg bag and some amount of preparation is required before we take him. So, I would say in his case, I would say that I prefer the telephone conversations and the virtual one, because he has a mobility issue* (Tarala, female, 78 years old).

Contrarily, reflecting on their own experiences of struggling with virtual care, Irfaan notes,

*I can’t express myself in English. You can maybe say one or two things but can’t do a proper conversation in English. English is harder over call without body language too. In person is better. Immigrants like us have the most trouble with this. They can’t say things properly* (Irfaan, male, 68 years old).

Similar to Irfaan, other participants shared the perspective that virtual care could be advantageous for older adults who have a strong command of the English language. However, they also noted that it could present difficulties for certain groups such as recent immigrants, emphasizing the importance of body language in facilitating communication when language barriers exist. Zarina also offers insights based on similar experiences with virtual care.

*“Doctor samne nahi hota hai toh baat hi nahi hota. Adhi bimari doctor seh baat karne se hi khatam ho jata hai” [translation: if the doctor is not in front of me then engaging with them does not feel the same. Half my sickness goes away when I speak to the doctor face-to-face]. Covid ruined everything. Virtual care was not satisfying. You forget what you needed to say in the middle of it and can’t reach them again after. In person conversations with my doctor has more feeling, more interaction* (Zarina, female, 60 years old).

In contrast to the quotes mentioned earlier, Zarina discusses the disadvantages brought on by the pandemic and the reliance on technology instead of in-person appointments, strongly indicating that face-to-face interactions with healthcare providers were preferable. Other participants emphasized different issues connected to virtual care, specifically regarding privacy concerns. Rajib explains his perspective on it,

*I live with my son and daughter-in-law, so when I have to speak to a doctor over a computer or over the phone, I can’t do it alone. There isn’t any privacy in a household like this, we are all together and around each other all the time. It can be uncomfortable to talk about certain things in that environment. But in-person is just me and the doctor—I can just tell my son to wait outside the doctor’s office* (Rajib, male, 74 years old).

Other participants living in multigenerational households with their children and grandchildren in the study also echoed similar concerns regarding privacy in virtual care settings. Some participants also explained that discussing certain sensitive topics in front of family members of the opposite gender is deemed inappropriate or discouraged in their cultural and familial context.

### Culture competence and humility

In discussing the health care experiences and needs of South Asian older adults, most participants highlighted cultural competence and humility as necessary components of healthcare delivery to meet the needs of patients effectively and to ensure healthcare providers possess vital information about the patients’ health.

#### The need for cultural competence in health care provision or services

Participants in the study expressed varying opinions regarding the importance of culturally competent care, with some emphasizing its importance, while others expressing that it was not essential. Most participants predominantly linked the need for medical care to physical health issues and ailments and believed healthcare professionals did not necessarily need to be aware of their ethnocultural background. For example, Shankar provides his viewpoint on the significance of culturally competent care,

*They need to give me proper care and that’s about it. My culture may impact my personal health, like food or practices, but I need doctors to just provide good care and solve my ailments. That’s really it* (Shankar, male, 86 years old).

Shankar’s statement emphasizes the potential disparity in how individuals perceive health and healthcare. It suggests that patients prioritize the role of culture when it comes to their own well-being, but this aspect may not hold the same significance when considering the Canadian healthcare system as a whole. Participants largely emphasized the importance of cultural competence when it comes to addressing mental health needs. For instance, Janaia discussed her experience with her psychiatrist.

*My appointments with my psychiatrist were not helpful at all because she did not understand that I come from a different culture, and background. So, the discussions were based on a very, on a very sort of vague, you know, a vague understanding of how Indian people conducts oneself within a family setup. And I found she didn't understand my background and what it meant to be a 60 something year old person who comes from an Indian culture and race. At the end of the sessions, I'd feel frustrated. In the sense that I sit here, and we talk, but I don't even feel that you understand the basics of where I have come from. My psychiatrist was totally disconnected with my background* (Janaia, female, 64).

The quote demonstrates the significance of a healthcare provider adopting a culturally competent approach in this scenario. It highlights the importance of understanding the patient’s familial structure and dynamics, as without this understanding, any medical guidance provided to the patient may lack relevance. Other participants in the study described how cultural competence could be a relevant factor even in addressing physical health requirements. When asked about why it might be important for her doctor to have or inquire about cultural understanding in relation to her physical health, Aileen said responded by stating,

*I think it's important for doctors to understand our family structure, and how we operate within that family structure. My doctors say we have to have a structure in our lives where we can have two to three hours of physical exercise a week for ourselves to get out to go for a walk, listen to some music, meditation, things like that. Giving yourself time going out, you know, perhaps for a coffee with your friends. That's all very well, it's all very good—in theory, we'd all like to do that. But I today live in this house, this home with just my husband and myself. And it is a struggle for me to get out and dedicate three to four hours a week for myself. Who runs the home? All that domestic chores. And this is not only unique to our culture, even within the Western culture is the woman who takes on more than the man does, right? The expectations are different. I have to be the one to cook and put dinner on the table every day, not my husband or my children or my in laws. It’s just how it works in our culture. So now if my family doctor says go exercise or go take time for yourself without understanding what responsibilities I have, do you think I can follow through?* (Aileen, female, 68).

Aileen’s quote emphasizes the importance of adopting a culturally tailored approach even when addressing aspects related to physical health, such as exercise prescription. The quote also brings attention to the intersectional experiences of participants in our study, specifically those who identify as racialized individuals and women.

#### Holistic vs. biomedical model of care

Participants emphasized that there exists a lack of acceptance towards any form of medication or healing practices that fell outside the boundaries of allopathic medicine. As an example, Beena shares her experience of her primary physician disregarding her suggestions regarding ayurvedic medications,

*I had severe pain due to some nerve problems and they completely disregarded the pain I was feeling after I tried the medications he prescribed. So, I suggested ayurveda to help with this, and he was so dismissive of that. And then when the man wasn't taking me seriously, I was seriously upset, and I'm not fussy like, he knows me well enough to know, I'm not a hypochondriac. But he wasn't supportive. And it came to a stage where I didn't even bother telling him certain things. I just did my own thing. I basically felt I had to advocate for myself* (Beena, female, 73 years old).

Beena explains how she addressed her doctor’s resistance towards Ayurvedic medicine through self-advocacy. This example also highlights the reluctance of healthcare providers to be receptive and knowledgeable about alternative healing practices that deviate from allopathic medicine.

One of the participants in our study, Sharad, who is a retired healthcare professional, provided an explanation for the necessity of a culturally competent approach when engaging with ethnocultural communities.

*As a provider, I would like to know what their perspective is on health and taking care of their body. I'd like to know, what is their indigenous method of treatment, home treatment that they use? And what do they do? Because people have traditional methods that they bring with them. And unless you understand that, well, maybe your Medicare treatment may be interfering with that, and you know, you may have problem without realizing what is happening, you know, so it's important to not only understand it, but explore it, educate yourself in that method, whatever it is, you know, it may be traditional Chinese medicine, it may be Unani, it may be Ayurveda. It may be homeopathy that comes from Europe, or Germany. It may be Native American treatment, depending upon who you're treating. You got to know what it means, and you have to be open to it. You can't ask somebody to do this or that, you know, without understanding what they are, they're limited to, or what they will do, there may be something richer, under the skin, you know? Very important that you don't just dismiss it* (Sharad, male, 87 years old).

Sharad’s perspective emphasizes an additional aspect of the significance of cultural competence, which lies in healthcare providers’ awareness of the potential treatments and medications employed by their patients, which is essential to prevent contraindications and ensure optimal healthcare outcomes. This viewpoint underscores that the significance of cultural competence extends beyond showing respect for others and their cultures as it also holds medical utility and practical value. It also highlights that healthcare providers need to be open to approaches to health and healthcare that are not white-centric.

### Negotiating candidacy: the need to “fit in”

Koehn (128) argues that older immigrants negotiate their “candidacy,” which refers to how people assess their eligibility to access healthcare services and how they justify their relationship with and use of services. The process of effectively presenting oneself, in order to establish the legitimacy of a claim to candidacy is challenging for both immigrants and older people in general (128). The ability of older immigrants to make a legitimate claim is often constrained by language inconsistency between care provider and recipient (128, 129). According to Koehn (128), the practice of othering becomes apparent when healthcare practitioners categorise patients and subsequently refer them to screening and expert services based on the patient’s presentation (or candidacy) and their own presumptions, professional or otherwise. The candidacy framework highlights the difficulty in gaining access to healthcare, as well as the need for a variety of measures to overcome obstacles faced by racialized immigrant older adults. The findings of our research suggest that the participants in our study have experienced a desire to conform to societal norms that have traditionally been established and maintained by white individuals.

#### The need to “fit in” and whiteness as a standard in healthcare

Participants in this research have recognized the need to modify their behavior and conform to the dominant Canadian system, including the healthcare system, in order to feel accepted and integrated. For instance, Shujata provided her viewpoint on why she was able to avoid experiencing discrimination as a patient within the Canadian healthcare system.

*I didn’t face that [discrimination] since I came here. Luckily. And that’s probably because of my personality and because I can speak the language very well. And that’s why the healthcare providers would not do these things with me. But if some other woman was there who doesn’t speak the language properly or wear the right clothes, that don’t fit the culture here, then those people will be underestimated and then treated like that, I think* (Shujata, female, 77 years old).

The quoted statement implies that the responsibility of being a “proper” citizen and consequently being able to access the healthcare system—a fundamental human right—rests on the patient. Shujata emphasizes that fluency in English, wearing appropriate attire, and feeling the need to assimilate into Canadian culture are requirements for accessing the healthcare system without encountering racial discrimination. Similarly, when asked about the importance of healthcare providers taking into account a patient’s ethnocultural background and its potential influence on their health, Aarini provided the following response:

*Religion is not even an issue that’s considered. Same for ethnicity and other things. When we all came 40 years ago, we had to be Romans in Rome. That means where we didn't have a lot of ethnic doctors available and things like that. So, we learn to live together with what we have. Immigrants, you know, when in Rome, act like Romans!* (Aarini, female, 62 years old).

Aarini further reinforces the notion of “assimilating” into Canadian society as a prerequisite for effectively using the healthcare system. Participants similarly also highlighted that the significance of religion and ethnicity is often overlooked since it is not as prominent of a factor in the considerations of Canadian society. Jamal offers an alternative viewpoint on how being an immigrant has not affected him and suggests measures that should be taken to prevent such impacts,

*No, we haven’t been impacted for being an immigrant because we don't cross our limits. We know there’s this border and we can't cross this border, because past that is danger. We stay within our bounds, and we stay with our own. We Pakistanis here think that way. One other example of this is that we are terrified of the police. They are strict on us. So, we don’t act in any way that will get us into trouble. We have to remember to stay within our bounds because we are immigrants, we are not like the Canadians here* (Jamal, male, 60 years old).

Jamal’s description of an implicit “border” that should not be crossed highlights the expectation for immigrants to assimilate and conform to a predetermined set of norms. According to him, there seems to be an unspoken rule for immigrants to adhere to specific behaviors and conform to certain societal expectations, both their own and that of their new society. Jamal further explained that this need to adhere to specific expectations extends to the healthcare context; for instance, one such rule that people must adhere to is speaking English or arranging for someone (e.g., children) who speaks English, in order to navigate and access the healthcare system.

A prominent and recurring theme in the interviews with the participants revolved around the concept of “whiteness” and white individuals being regarded as the norm or standard. Although the participants acknowledged that whiteness should not be regarded as the standard, they explained that this internalized belief is held by both white individuals and those from racialized backgrounds. One of the participants, Srijit, explains this phenomenon succinctly,

*New immigrants from India and Bangladesh could be intimidated by a white doctor, white Caucasian doctor. And we seem to have this thing that you know, we always respect to the highest degree to the Caucasian people. I see that it's basically a reverse discrimination that we do, in fact. Our mind is wired for white supremacism—we are dark, and they are white so let us elevate this class up there. But this should not be so* (Srijit, male 75 years old).

Additionally, participants emphasize that the feeling of being considered “lesser than” sometimes leads to a lack of belongingness in their current communities compared to their countries of origin. On the issue of assimilating into a Canadian society that puts whiteness and white individuals at its center, Janaia discusses the influence it has on her sense of identity and consequently, her health and overall well-being.

*I moved to Canada in the 80s. When it was not what it is today. It was still very much a white country, more so than now. You know, to fit in, to fit in, was hard. I was one person at home and another person outside of the home. And it created that dual identity for myself. And I feel that at this stage now, I feel that is so detrimental to an individual. Because you, you're not one or the other. Right? And who are you as a whole? You know, and I have brought the best from the Western culture to my own, but at the same time, it has impacted my own ethnic culture. Where do I fit in with the ethnic culture? Do I one hundred percent fit into my ethnic culture? No, I don’t. Do I do I fit in with the Western culture one hundred percent? No, I don't. So here I am. I'm a whole of two different cultures. And you can say that, you know, it's, enhanced me, but I would say, perhaps, it has been detrimental to me* (Janaia, female, 64 years old).

Janaia’s statement emphasises the complex nature of identity experienced by racialized immigrants living in a predominantly white country such as Canada. It brings attention to the dualities they face, which can have adverse effects on their well-being and sense of belonging. It is important to note that viewpoints such as the one above was provided by participants, spontaneously, during a discussion where they were prompted to reflect on their heath as an immigrant and their healthcare experiences in Canada. Participants’ quotes, therefore, reflect not only their specific healthcare experiences but also their perspectives on the sociopolitical system at large that they believe impacted their health and healthcare experiences at the individual, interpersonal, level.

## Discussion and recommendations for policy and practice

### The need to look at broader society to understand individuals’ experiences

Sociologist Emile Durkheim once stated that, “one can understand people, not by looking at individuals, but only by examining their society” (130). In the context of our research, this idea implies that one must consider the society to which racialized immigrants belong, including the factors that shaped its current state and the individuals or groups responsible for its formation. By doing so, we can gain a deeper understanding of racialized immigrants’ experiences and the broader social dynamics at play that impact those experiences (23). The issue of access is such that it necessitates a comprehensive understanding at both the systems level and the interpersonal level for an adequate understanding of its complexities (131). Although considerable efforts have been made to offer adequate healthcare services to all Canadians, disparities persist in the utilization and access of primary healthcare and preventative services between foreign-born and Canadian-born residents (132–134). Various factors, such as geographical factors, economic constraints, limited operating hours, wait times, language barriers, challenges in navigating the healthcare system, and cultural differences, present significant obstacles to accessing healthcare services (135–138). Additionally, studies have found factors such as relying on others to take time off for appointments (139), transportation issues (138, 140), the need to prioritize household responsibilities over health concerns (140), and delaying seeking healthcare until it becomes urgent (136, 138), can hinder new immigrants’ access to healthcare. Results of our study corroborate these findings. For example, participants emphasized factors such as depending on family members for appointments and not having enough personal time for self-care and health-related pursuits (such as exercise) due to their familial responsibilities. Consistent with our findings, Attum et al. (141), whose study focused specifically on Muslim patients, affirm that the aim of understanding how to provide care for patients is to empower all healthcare professionals with the capacity, knowledge, and skills to meet the distinct needs of each patient and, just as importantly, their family. The findings of this study, therefore, indicate the necessity for healthcare providers, and the larger healthcare system, to refrain from making assumptions about familial dynamics (141, 142). It is crucial to abandon the notion that all families operate in the same manner (143). Instead, a personalized approach is needed to offer culturally and individually suitable medical and healthcare advice to patients (143–145). As highlighted by our participant, Aileen, failure to consider such nuances of familial structure and individual contexts could lead to overlooking underlying factors that may significantly impact patient care (146). It is, therefore, important to implement policies that focus on cultivating cultural competence and humility, at the systems level, for those entering or currently in the healthcare and medical field (147). To achieve this, it is vital to avoid merely tokenistic or performative gestures of cultural awareness, but rather adopt actionable, result-oriented, steps to ensure cultural safety at the organizational and systemic level (148, 149). Effective approaches may involve making cultural safety an essential criterion for accreditation and continuous certification of healthcare institutions and practitioners (150, 151). Additionally, mandating cultural safety training and performance evaluation for all staff, supervisors, and assessors could also prove helpful in achieving this goal (151).

### The need for translators and interpreters

The literature reports that language services are a critical aspect of access to care for racialized immigrants (152–154). de Moissac and Bowen (155) report that immigrants facing language barriers were at a higher risk of encountering medication errors, delays in diagnosis, and suboptimal care. Additionally, immigrants were more prone to having a strained relationship with their healthcare providers, facing challenges in effectively communicating their health needs, developing mistrust, and experiencing racism and discrimination (155–158). These findings were consistent with the current study, as participants emphasized that language barriers and the absence of language or translation services hindered healthcare access. This study underscores the importance of offering language assistance during medical consultations to address language barriers faced by immigrants. To ensure high-quality, equitable, and patient-centered care, this study supports the establishment of guidelines for recruiting, training, and effectively engaging language interpreters during medical consultations (153, 159–163). Participants in this study, similar to findings in other research studies (153, 161, 164, 165) emphasized the necessity of having bilingual healthcare providers. Molina and Kasper (166) advocate for language-concordant care, as it has been shown to offer safe and high-quality healthcare. However, the findings of our study suggest caution against assuming that care will automatically be of good quality solely based on language concordance. Additionally, echoing Shommu et al. (167), community health navigators can play a vital role in improving access to primary and preventative healthcare services and can act as cultural brokers and language interpreters for racialized immigrant older adults. Lastly, recognizing that implementing cultural competence/humility and having medical interpreters available in every healthcare sector in Canada might be an ambitious idea, the study’s findings propose that these aspects could hold greater significance in certain sectors than others. Cultural competence/humility and medical interpreters may play a more crucial role in mental healthcare services compared to acute care settings (83). As an example, the presence of a medical interpreter and cultural competence/humility may hold greater significance when a patient seeks mental health support, necessitating the explanation of intricate family dynamics, as opposed to the role of these services during routine blood work at a clinic.

### Considerations related to virtual care

Our results showed that virtual care could both facilitate and impede access to healthcare services for South Asian participants. Much like Ahmed et al. (138), pointing out transportation challenges as a hindrance to factors, some of our participants noted that virtual care was advantageous because it provided convenience, especially for individuals with disabilities, eliminating the need to travel to see a healthcare provider. However, other participants expressed concerns that virtual care created a greater divide from their already distant (i.e., hard to reach) healthcare providers. Additionally, virtual care brought forth other challenges, such as a lack of privacy, which hindered patients’ ability to access healthcare providers in a confidential and sensitive environment. Hardcastle and Ogbogu (168) also highlight privacy issues in virtual care, emphasizing that irrespective of the delivery method, adherence to privacy laws is imperative for virtual healthcare services. In our study, participants primarily emphasized privacy concerns at the interpersonal level, mentioning their reluctance to discuss sensitive health matters in front of their children or grandchildren, who, in multi-generational households, often help with the set-up of virtual appointments. In contrast, Hardcastle and Ogbogu (168) addressed privacy from a broader perspective, examining aspects such as the ownership and custodianship of virtual care health information, broad authorization for unspecified use and sharing of patient health information, as well as data security. Regarding privacy concerns at the interpersonal level, Cohen and Casper (169) offer valuable insights related to racialized communities, particularly Black and Latino individuals. Their research relates the arrangement of multigenerational households and its implications to privacy issues as well as individual independence (169). However, while our study addresses the privacy of older adults living within their children’s homes, Cohen and Casper (169) examine the privacy concerns of younger adults who depend on the support of their older relatives. Even though virtual care saw rapid adoption in response to COVID-19, there is now an opportunity for policymakers to reassess its integration before it becomes a permanent fixture in the healthcare system (168, 170, 171). Beyond regulatory considerations, policymakers and healthcare providers should also address the specific privacy concerns of racialized immigrant older adult populations living in multigenerational households. Taking all these factors into account will contribute to a more thoughtful, effective, and culturally competent approach to virtual care implementation (172).

### Whiteness as the standard, and making space for deviations from the standard through cultural competence and humility

The fundamental concept of white normativity is succinctly described by Morris (173) in the following manner: white individuals are considered fully human, while individuals from other racial groups are deemed fully human only to the extent that they resemble white individuals. Although it can be intertwined with explicit discrimination or racial animosity, white normativity operates in a more nuanced manner (173). Whiteness establishes the standard or accepted spectrum of behaviors and traits, and all other racial groups are compared against whiteness as deviations from this norm (174–176). Consequently, whiteness occupies a central position in the classification of races and the process of racialization (173, 177).

As the recognition to dismantle white normativity grows, both in Canada and globally, there is an increasing emphasis on the significance of establishing an equitable healthcare system (178). However, Canada’s models of care operate with the assumption that biomedical model of health rooted in Eurocentric traditions of medicine is the all-encompassing gold standard (179, 180). The healthcare system in Canada, therefore, is structured around Eurocentric approaches to health and well-being (181, 182). However, such an approach ignores a significant portion Canada’s population that are racialized. Participants of our study expressed that their approach to well-being and understanding of it did not align with their healthcare providers, resulting in unmet health needs. In relation to seeking medical advice for her mental health, one of our participants highlighted that the recommendations provided by her psychiatrist were not suitable for her circumstances as a female Indian immigrant residing in a multi-generational household. A culturally competent approach would require the provider to understand the context of a patient’s life and the cultural underpinnings that affect it (183). Additionally, a culturally humble approach requires that a healthcare provider does not take a standardized prescriptive approach, but rather one that tailors care to their unique context. This means that healthcare providers need to move away from the Eurocentric approach that places white people in the centre and make space for other ways of knowing and being that go beyond Eurocentric traditions of medicine. Medical discourse in the Global North should be more accepting of traditional medicinal knowledge and health practices that generations of racialized and immigrant communities have transmitted and reproduced (184). Analogous to how whiteness is the standard, and non-whiteness a deviation, “Western” medicine is seen as the convention, and any “alternative” form of knowledge is unconventional and a departure from the norm (65). Medicine in the Global North is largely based on biomedical foundations of health and well-being, while several definitions of health in the Global South take a more holistic approach (62, 185, 186). For instance, while medicine in the Global North primarily emphasises clinical care, participants in this study highlighted approaches such as Ayurvedic medicine, which revolves around the idea of fostering well-being instead of merely combatting illnesses and upholding a harmonious balance between the individual and nature (187–189). Forms of healing that originate in the Global South often diverge away from aspects of medicine in the Global North rooted in technological, biomedical, and pharmaceutical hegemony (65). Therefore, it is important for the healthcare system to recognize the legitimacy of other ways of “knowing,” and therefore, healing.

### Negotiating candidacy: fitting the mould of whiteness or shedding difference?

The Healthy Immigrant Effect posits that immigrants have a health advantage over Canadian-born citizens that diminishes with length of residency (190, 191). These variations underscore the need to identify if and how older immigrants encounter barriers related to healthcare access (192). Although the older population in Canada is growing and its ethnic makeup is rapidly diversifying, racialized immigrant older adults are underrepresented in research and in social and health policies because it is assumed that they are cared for by their families and that their numbers are too small to be considered a “problem” (193). According to Brotman (194), the strength of filial piety is often referenced by healthcare providers as a way to shift the responsibility of care onto family members, which can have detrimental effects on both caregivers and older adults. Othering is a pattern of differentiation and marginalisation based on identities that depart from the norm, impacting how individuals perceive and treat those who are viewed as belonging to the “in-group” as opposed to those who are viewed as belonging to the “out-group” (194–196). In our study, participants specifically detailed the occurrences of othering they experienced, which involved the dismissal of healing practices from their cultural heritage, such as Ayurveda. Participants also expressed the impact of othering that stemmed from internalized white normativity. In the context of healthcare, othering acts as discrimination and a barrier to care access, rooted in institutional structures and policies that were intended to be equitable but created using white-centric ideologies and frameworks (151).

As a consequence of employing approaches and health care practices deeply entrenched in the Eurocentric paradigm, both at the interpersonal and systemic levels, individuals who are non-white are expected to conform to these pre-established principles (46, 197). Consistent with existing literature, our research findings indicate that the individuals we spoke to expressed an inclination to conform to societal norms that have historically been set by white individuals but are upheld by both white individuals and racialized persons who have internalized white normativity and those who are so deeply colonized that they discipline themselves, and each other, for stepping outside of the standard template. Koehn’s (128) candidacy framework aligns well with the findings of this study since it highlights the challenges faced by racialized immigrant older adults in accessing healthcare and emphasizes the requirement for ethnoculturally diverse individuals to overcome these obstacles. As an example, participants in this study discussed the necessity of dressing in a manner that resembles white individuals to receive suitable healthcare services from healthcare providers. These findings are consistent with other research in the literature that has shown that clothing choices and adopting speech patterns similar to those of white individuals decreases racial stereotyping and act as a protective measure against discrimination (198–205). Thus, our research, consistent with existing literature, reinforces the notion that racialized immigrant communities are subjected to certain expectations and constraints to meet the standards of whiteness, in order to attain the rights and privileges granted to white individuals, as a condition of being considered “good” citizens (206).

### Intersectionality

Despite the increasing usage of the term “intersectionality” in academic discourse, researchers often oversimplify and overlook the interconnectedness of people’s multiple identities (207). The findings of this study highlight that the category of “older adults” is significant, considering the biological changes that occur during this phase of life, necessitating modified medical interventions (208). However, it is important to understand the “older adult” category in relation to those who also encompass diverse identities and cultural connections that they have cultivated throughout their lives (209). It is simultaneously also important to recognize that these identities, along with personalities, habits, and preferences, persist and evolve throughout one’s lifespan, including into older adulthood (207, 210).

The current study also supports the notion that micro-level factors, such as an individual’s racial or ethnocultural identity, and macro-level factors, such as colonial history, are interconnected and present in every social interaction (211). An individual’s race might be relevant to or even dominate how they experience a given social interaction or circumstance, but there may also be other identities at play that are less obvious (212). Therefore, we must be aware of and open to the possibility that other axes of inequality, interwoven inseparably with race, are at work in addition to race as the governing explanatory factor for a given experience (211, 212). Race, racialization, or racism cannot be considered the sole explanatory factor for any given experience (212). Therefore, intersectionality affirms that race, class, gender, religion, and other social constructs are mutually constitutive, dynamic, and flexible categories that operate with one another dynamically and in varying degrees of tension (i.e., individual, systematic, structural, institutional, global) within broader social environments and in the context of healthcare (40, 211).

Lastly, it is important to acknowledge that at the forefront of this study concerning racialized immigrants lies the intersection of white normativity, patriarchy, and capitalism as the underlying factors influencing global migration patterns (53). These forces significantly mold the encounters of people of the global majority in both migrant-sending and receiving communities (53, 213, 214). Therefore, it is essential to bring about significant and lasting improvements in the health and well-being of racialized individuals on a global scale (215). This requires a thorough and critical examination of the social position of white people regarding their role in the historical scientific enterprise, as well as their influence on power dynamics (216). Understanding the reasons behind the current state of affairs also requires recognition of contemporary colonial practices, and the implementation of top-down global health governance and programming initiatives (217).

## Limitations

Our qualitative study has some limitations. First, our sample of participants was geographically limited because we recruited participants from Southern Ontario, focusing primarily on urban cities. This also resulted in the possible exclusion of racialized immigrant older adults residing in rural areas who may have a lower socio-economic status. Secondly, although we conducted in-depth interviews with older adults to explore their perspectives on systemic barriers and how it impacts healthcare experiences, our study would benefit from additional insights by also interviewing family caregivers about their role in providing care. Similarly, it would have been beneficial to interview healthcare providers regarding their firsthand experiences in engaging racialized immigrant older adults and their families in care, providing insights from the perspective of care providers. Thirdly, this study focused on only on the experiences and perspectives of the South Asian immigrant communities in Canada. Subsequent research should investigate the viewpoints and experiences of other communities within the Canadian demographic landscape, such as the Chinese community and the Arabic-speaking community. Fourthly, we had a large number of participants who were highly educated, came from higher socioeconomic backgrounds, and had high English language proficiency. Although our sample aligns with the migration patterns of South Asian older adults in Canada (i.e., many South Asian families migrated to Canada during the mid-20th century and are now older adults, as reported by the Government of Canada in 2021 (218)), it is important to understand and specifically study the experiences of recent immigrants from less privileged socioeconomic backgrounds and those for whom English is a second language. Lastly, it is important for studies of this kind to specifically address refugees who have migrated to Canada, distinguishing between immigrants and refugees due to their significantly distinct experiences of migration.

## Conclusion

The objective of this study was to evaluate the structural determinants and systemic factors influencing the healthcare experiences and overall health and well-being of South Asian older adults in Canada. Consistent with previous literature, the study found that challenges with accessibility remains a critical issue for racialized immigrant older adult populations (132–134), while the rapid adoption of technology presents novel challenges that demand attention in both healthcare research and practice (168). To tackle these issues related to accessibility and address the need for reform healthcare at a systems level, person-centered care and cultural competence and humility are proposed as potential solutions (83). Additional research is necessary to devise innovative solutions for addressing the current challenges encountered by the racialized immigrant older adult population. For instance, exploring the potential of virtual care platforms to facilitate language and translation services could prove beneficial. Implementing such solutions at a provincial and federal level becomes especially important and relevant given the increasing number of immigrants in Canada and the growing integration of technology in healthcare practices.

The findings of the study support the notion that intersectionality can be utilized to explore the unique interactions between experiences such as education, immigration status, age, and gender and how they are positioned within larger oppressive systems (34). The impact of a colonial and oppressive history continues today with perpetuating racism that is ubiquitous in our societies, deeply entrenched at the institutional and interpersonal level (5, 219). It is, therefore, essential to start considering racism as a determinant of health (215). Health researchers, practitioners, and decision-makers in Canada should not shy away from addressing the complexities of racism in combination with other “isms” (220, 221). To cater to the needs of racialized immigrant older adults, it is crucial to offer a diverse array of healthcare options, including “non-Western” systems that have been and continue being “othered” (196, 222). Embracing diversity and finding innovative solutions to longstanding healthcare challenges requires challenging the prevailing dominance of the biomedical model of health and research (223, 224). It is also important to move beyond an exclusive emphasis on assimilation theories, since they center around whiteness and focus on how immigrants of color become more similar or distant from the dominant group, middle-class white people, over time (225–230). Anti-oppression and anti-racism frameworks can aid in identifying, analyzing, and transforming the attitudes, structures, and behaviors that perpetuate systemic racism, white normativity, and other forms of societal oppression (231). It is equally important to establish best practices, incorporating both cultural competence and humility frameworks, to effectively serve a culturally, ethnically, and racially diverse population (232). Systemic structures in Canada contribute to inequitable and biased systems, making it imperative to establish frameworks that receive adequate funding and systemic support to address the considerable challenge of rectifying historical injustices and acknowledging ongoing ones.

## Data Availability

The datasets presented in this article are not readily available because due to the nature of the research and ethical constraints, supporting data is not available. Requests to access the datasets should be directed to dchowdhury@uwaterloo.ca.
